# Supplementary material for: Robust inference of kinase activity using functional networks
Source: Nat Commun. 2021 Feb 19;12:1177. doi: 10.1038/s41467-021-21211-6 (PMC7895941; doi:10.1038/s41467-021-21211-6)
Supplement: Supplementary file 2 — Description of Additional Supplementary Files [file 41467_2021_21211_MOESM2_ESM.pdf]

# Robust Inference of Kinase Activity Using Functional Networks

## Description of Additional Supplementary Files

### Supplementary Data 1 - 25 Annotated Kinases

Supplementary Data 1 contains information about the 25 kinases that have at least one annotation for the 80 perturbations analyzed in our study. The file contains a data matrix with 7 columns which are given as follows:

1. Ensembl ID
2. Uniprot ID
3. Kinase Name
4. Gene Symbol
5. Description of the kinase
6. Number of perturbations the kinase is annotated in (out of 80)
7. Number of substrates of the kinase (according to PhosphositePlus data)
