# Supplementary material for: Robust inference of kinase activity using functional networks
Source: Nat Commun. 2021 Feb 19;12:1177. doi: 10.1038/s41467-021-21211-6 (PMC7895941; doi:10.1038/s41467-021-21211-6)
Supplement: Supplementary file 4 — Reporting Summary [file 41467_2021_21211_MOESM4_ESM.pdf]

nature research

Corresponding author(s): Serhan Yılmaz

Last updated by author(s): Dec 15, 2020

Reporting Summary

Nature Research wishes to improve the reproducibility of the work that we publish. This form provides structure for consistency and transparency in reporting. For further information on Nature Research policies, see our Editorial Policies and the Editorial Policy Checklist.

Statistics

For all statistical analyses, confirm that the following items are present in the figure legend, table legend, main text, or Methods section.

n/a	Confirmed	
☐	☒	The exact sample size (*n*) for each experimental group/condition, given as a discrete number and unit of measurement
☒	☐	A statement on whether measurements were taken from distinct samples or whether the same sample was measured repeatedly
☐	☒	The statistical test(s) used AND whether they are one- or two-sided<br>*Only common tests should be described solely by name; describe more complex techniques in the Methods section.*
☒	☐	A description of all covariates tested
☒	☐	A description of any assumptions or corrections, such as tests of normality and adjustment for multiple comparisons
☐	☒	A full description of the statistical parameters including central tendency (e.g. means) or other basic estimates (e.g. regression coefficient) AND variation (e.g. standard deviation) or associated estimates of uncertainty (e.g. confidence intervals)
☐	☒	For null hypothesis testing, the test statistic (e.g. F, t, r) with confidence intervals, effect sizes, degrees of freedom and P value noted<br>*Give P values as exact values whenever suitable.*
☒	☐	For Bayesian analysis, information on the choice of priors and Markov chain Monte Carlo settings
☒	☐	For hierarchical and complex designs, identification of the appropriate level for tests and full reporting of outcomes
☐	☒	Estimates of effect sizes (e.g. Cohen's *d*, Pearson's *r*), indicating how they were calculated

Our web collection on statistics for biologists contains articles on many of the points above.

Software and code

Policy information about availability of computer code

Data collection	In this study, we only used publicly available datasets from previous studies. Therefore, no software was used for data collection.
Data analysis	We used custom algorithms and software that we implemented in Matlab for data analysis. These custom code are provided with the submission, and the software is deposited to Github (https://github.com/serhan-yilmaz/RoKAI). Note that, the structure of the custom code used for the analysis is illustrated in the provided 'analysis_code_schematic.pdf' document.

For manuscripts utilizing custom algorithms or software that are central to the research but not yet described in published literature, software must be made available to editors and reviewers. We strongly encourage code deposition in a community repository (e.g. GitHub). See the Nature Research guidelines for submitting code & software for further information.

Data

Policy information about availability of data

All manuscripts must include a data availability statement. This statement should provide the following information, where applicable:
- Accession codes, unique identifiers, or web links for publicly available datasets
- A list of figures that have associated raw data
- A description of any restrictions on data availability

We obtain the benchmarking data from publicly available datasets of previous studies (Ochoa et al., 2016; Hernandez-Armenta et al., 2017, http://phosfate.com/download.html). We obtain the kinase-substrate annotations from PhosphositePlus (http://www.phosphosite.org/staticDownloads). We obtain the human protein-protein interaction network from STRING (Szklarczyk et al., 2014, http://string-db.org/cgi/download.pl). We obtain the co-evolution and structure distance evidence between phosphosites from PTMcode (Minguez et al., 2012, http://ptmcode.embl.de/data.cgi). We obtain the predicted kinase-substrates edges by NetworKin (Horn et al. 2014, https://networkin.info/download.shtml). The materials (code and data) to reproduce the results are available in figshare with the identifier: https://doi.org/10.6084/m9.figshare.12644864.

Field-specific reporting

Please select the one below that is the best fit for your research. If you are not sure, read the appropriate sections before making your selection.

- [x] Life sciences
- [ ] Behavioural & social sciences
- [ ] Ecological, evolutionary & environmental sciences

For a reference copy of the document with all sections, see nature.com/documents/nr-reporting-summary-flat.pdf

Life sciences study design

All studies must disclose on these points even when the disclosure is negative.

Sample size	This study presents computational experiments on publicly available data. We used all the data available and we have investigated the effect of missing data as well. In other words, the results presented in the paper provides information on the effect of sample size and missing data.
Data exclusions	We excluded the perturbation described as "PIK3CA activation (H1047R) + inhibitor" from our analysis because the available annotations for upregulated and downregulated kinases in this perturbation could be unreliable since this perturbation is a mixture of both an activator and an inhibitor. This was a pre-established criteria to improve the quality of our results.
Replication	All experiments that are reported are computational and all scripts that are used to generate the results are available (which can be used to replicate our results). In addition, since the nature of our analysis is computational, we performed 100 simulation experiments (where a portion of the available annotations are hidden from the inference methods) to assess the robustness and replicability of our results. Overall, we observed consistent results across these 100 simulation experiments, thus, we consider our attempts at replication successful.
Randomization	Randomization was only used in computational simulations of missing data (on kinase-substrate annotations). In that case, the data to be removed was selected uniformly at random and the experiments were repeated 100 times. We calculated and reported confidence intervals for all of these computational experiments.
Blinding	The aim of the method is to predict dysregulated kinases. In our computational experiments, the dysregulated kinases are blinded or all prediction algorithms that are considered in an unsupervised manner.

Reporting for specific materials, systems and methods

We require information from authors about some types of materials, experimental systems and methods used in many studies. Here, indicate whether each material, system or method listed is relevant to your study. If you are not sure if a list item applies to your research, read the appropriate section before selecting a response.

Materials & experimental systems

n/a	Involved in the study
[x]	[ ] Antibodies
[x]	[ ] Eukaryotic cell lines
[x]	[ ] Palaeontology and archaeology
[x]	[ ] Animals and other organisms
[x]	[ ] Human research participants
[x]	[ ] Clinical data
[x]	[ ] Dual use research of concern

Methods

n/a	Involved in the study
[x]	[ ] ChIP-seq
[x]	[ ] Flow cytometry
[x]	[ ] MRI-based neuroimaging
